# Genetic Screening of Mutations Associated with Fabry Disease in a Nationwide Cohort of Juvenile Idiopathic Arthritis Patients

**DOI:** 10.3389/fmed.2017.00012

**Published:** 2017-03-01

**Authors:** Maria J. Gonçalves, Ana F. Mourão, António Martinho, Olívia Simões, José Melo-Gomes, Manuel Salgado, Paula Estanqueiro, Célia Ribeiro, Iva Brito, João E. Fonseca, Helena Canhão

**Affiliations:** ^1^Rheumatology Department, Hospital Santa Maria, Lisbon Academic Medical Centre, Lisboa, Portugal; ^2^Rheumatology Research Unit, Instituto de Medicina Molecular, Faculdade de Medicina da Universidade de Lisboa, Lisboa, Portugal; ^3^Rheumatology Department, Centro Hospitalar de Lisboa Ocidental, Lisboa, Portugal; ^4^Centro de Histocompatibilidade do Centro, Coimbra, Portugal; ^5^Instituto Português de Reumatologia, Lisboa, Portugal; ^6^Pediatric Rheumatology Unit, Hospital Pediátrico de Coimbra, Coimbra, Portugal; ^7^Rheumatology Department, Hospital de Faro, Faro, Portugal; ^8^Rheumatology Department, Centro Hospitalar S. João, Porto, Portugal; ^9^EpiDoC, CEDOC, Nova Medical School, Lisbon, Portugal

**Keywords:** Fabry disease, pain, juvenile idiopathic arthritis, cohort, register

## Abstract

Fabry’s disease (FD) is a lysosomal storage disorder associated with an alpha-galactosidase A deficiency. The prevalence of FD among juvenile idiopathic arthritis (JIA) patients with established diagnosis is unknown, but as musculoskeletal pain may be an important complaint at presentation, misdiagnosed cases are anticipated. With this study, we aim to calculate the frequency of FD-associated mutations in a cohort of JIA patients. Children with JIA from a national cohort were selected. Clinical and laboratorial information was recorded in the Portuguese rheumatic diseases register (http://Reuma.pt). Molecular genetic testing to detect *GLA* gene mutations was performed. After the multiplex polymerase chain reactions technique for DNA amplification, direct sequencing of the complete sequence of *GLA* gene was completed. From a cohort of 292 patients with JIA (188 females, 104 males), mutations were identified in 5 patients (all female). Four patients had the mutation D313Y, a rare *GLA* variant, which is associated with low enzymatic levels in plasma, but normal lysosomal levels. One patient presented the missense mutation R118C, which was previously described in Mediterranean patients with FD. This is the first screening of FD mutations in a cohort of JIA patients. No “classic” pathogenic FD mutations were reported. The late-onset FD-associated mutation, R118C, was found in a frequency of 0.34% (1/292).

## Introduction

Fabry’s disease (FD) is a lysosomal storage disorder initiated by sphingolipid accumulation within lysosomes owing to an alpha-galactosidase A (alpha-Gal A) deficiency (1, 2). It is a monogenetic defect inherited in an X-related recessive manner (3). The defects in the GLA gene are heterogeneous, with over 649 mutations recorded according to The Human Gene Mutation Database (accessed 27th October 2015); the majority of these mutations are missense or nonsense (4). This enzyme defect generates progressive accumulation of globotriaosylceramide (Gb_3_) and related glycosphingolipids, primarily in the vascular endothelium (5).

Reported FD incidences range from 1 in 476,000 to 1 in 117,000 in the general population, and its estimated prevalence among males is around 1/40,000 (5, 6).

Classically, affected hemizygous males present with joint pain (60–80% of the patients), cutaneous lesions (*angiokeratomas*), and gastrointestinal (GI) symptoms (abdominal pain, diarrhea, and vomiting) (5, 7). Autonomic nerve system damage can appear early in the disease course, with patients reporting absence or decrease of sweating. Notwithstanding the absence of major organ dysfunction, these early symptoms, including arthralgia, can produce significant morbidity, limiting the quality of life of affected children (7).

During the course of the disease, more severe manifestations occur, including renal, cardiac, and cerebrovascular complications.

Contradicting a previous belief that women who are heterozygotic for disease causing mutations have a mild phenotype, an analysis from the Fabry Outcome Survey revealed that the majority of these females report clinical features of Fabry disease (8). Indeed, severe manifestations of disease were present, with 77% of women reporting neurological involvement, 59% cardiac involvement, and 40% renal involvement, although the onset of symptoms was not as early as is typically observed in males (9). A considerable delay between onset of symptoms and diagnosis in this group of women was also observed, underlining the importance of careful and longitudinal assessment of female heterozygote patients with FD (9). Recently, definite FD diagnostic criteria were defined, both for male and female patients (see Table S1 in Supplementary Material) (10).

Fabry’s disease is rare but causes high comorbidity and increases mortality. However, if early diagnosed can be treatable. Enzyme replacement therapy (ERT) with agalsidase beta has positive results, decreasing neuropathic pain (11) and reducing major organ complications (such as left ventricle hypertrophy) (12).

The screening of high-risk populations seems to be the best cost-effective strategy to diagnose new cases and intervene earlier. As musculoskeletal pain may be a chief complaint, children with joint pain may be considered a population at risk. Two types of pain in FD patients have been described: (1) episodes of burning pain in the extremities, radiating inwards, associated with low-grade fever; (2) acroparesthesias (chronic neuropathic pain in the extremities) (13, 14). These episodes tend to express early in the disease course (first and second decades of life) (8).

Juvenile idiopathic arthritis (JIA) is the commonest cause of chronic arthritis in childhood—0.07–4.01 per 1,000 children worldwide is affected (15). In the oligoarticular subtype (50–80% of all children with JIA), lower extremities are predominantly affected, with the knee being the most frequently involved joint, followed by the ankle (16). Also, in children and teenagers with enthesitis-related JIA, lower extremity pain is common (17). Furthermore, pain scores in Fabry disease were similar to those observed in a group of children with JIA (7).

The prevalence of FD among patients diagnosed as JIA is unknown. The correct diagnosis can prevent the progression of FD and avoid unjustifiable immunosuppression.

## Materials and Methods

We aimed to calculate the frequency of FD in a population of JIA patients by GLA genotyping. Carriers of Fabry’s-associated mutations were further characterized.

Children and young adults (mean age 17.7 years and 11 years of mean disease duration) from a JIA cohort of patients consecutively included from Paediatric Rheumatology centres in Portugal Mainland and Islands. All patients were diagnosed by an expert physician in the field and classified according to the International League of Associations for Rheumatology (ILAR) criteria (18). Clinical and laboratorial information was recorded at regular time points, according to clinical practice, in the Portuguese nationwide register http://Reuma.pt.

Additionally, a questionnaire with clinical information and a laboratorial evaluation of relevant data in the context of JIA were also performed in these patients. Blood samples collected have been stored at the IMM Biobank, Lisbon Academic Medical Centre.

This study was submitted to the Local Ethics Committee, at Hospital de Santa Maria, Lisbon Academic Medical Centre. For being included in the study, parents and patients gave their informed written consent.

Molecular genetic testing to identify *GLA* mutations was performed in the Centre of Histocompatibility in Coimbra. Direct sequencing was performed for samples from all patients in order to identify the precise mutation. Multiplex polymerase chain reactions with primers located in intron–exon boundaries were performed in order to allow sequencing of the complete coding region of *GLA* gene (seven exons). The *GLA* gene sequencing was performed in 3130 ABI sequencing platform (Applied Biosystems), and data were analyzed with SeqScape v3.0 and Geneious R9 software. Direct sequencing of the appropriate amplified fragment was a feasible strategy due to the development of rapid and sensitive automated sequencers. Control samples from healthy individuals were run in parallel with patients’ samples in order to confirm the consensus sequence for all the amplified exons.

## Results

Two hundred ninety-two patients (188 females, 104 males) from our JIA cohort were enrolled, including patients from Portugal Mainland and Azores and Madeira islands (Table 1). A female predominance was present (64.3%), and the mean current age and disease duration were 17.7 ± 9.2 and 11.0 ± 8.3 years, respectively. The most frequent forms of JIA (ILAR Classification) were oligoarticular (persistent and extended), comprising almost half of the patients, followed by polyarticular seronegative form (16.8%).

**Table 1:** **Clinical features of the JIA cohort**.

Cohort of JIA patients (*n* = 292)	(%)
Female gender	64.3
Age [mean (SD)]	17.7 (9.2)
Age at diagnosis [mean (SD)]	8.0 (5.0)
Disease duration [mean (SD)]	11.0 (8.3)
**JIA International League of Associations for Rheumatology classification**	
Persistent oligoarticular	33.0
Extended oligoarticular	15.8
Polyarticular RF−	16.8
Polyarticular RF+	8.9
Psoriatic arthritis	5.5
Enthesitis-related JIA	12.0
Systemic JIA	7.9
**Other features**	
Uveitis	13.7
Patients treated with cDMARDs	79.5
Patients treated with biologic DMARDs	20.2

*cDMARDs, conventional drug modifying anti-rheumatic disease drugs; JIA, juvenile idiopathic arthritis; RF, rheumatoid factor*.

**Figure 1:**
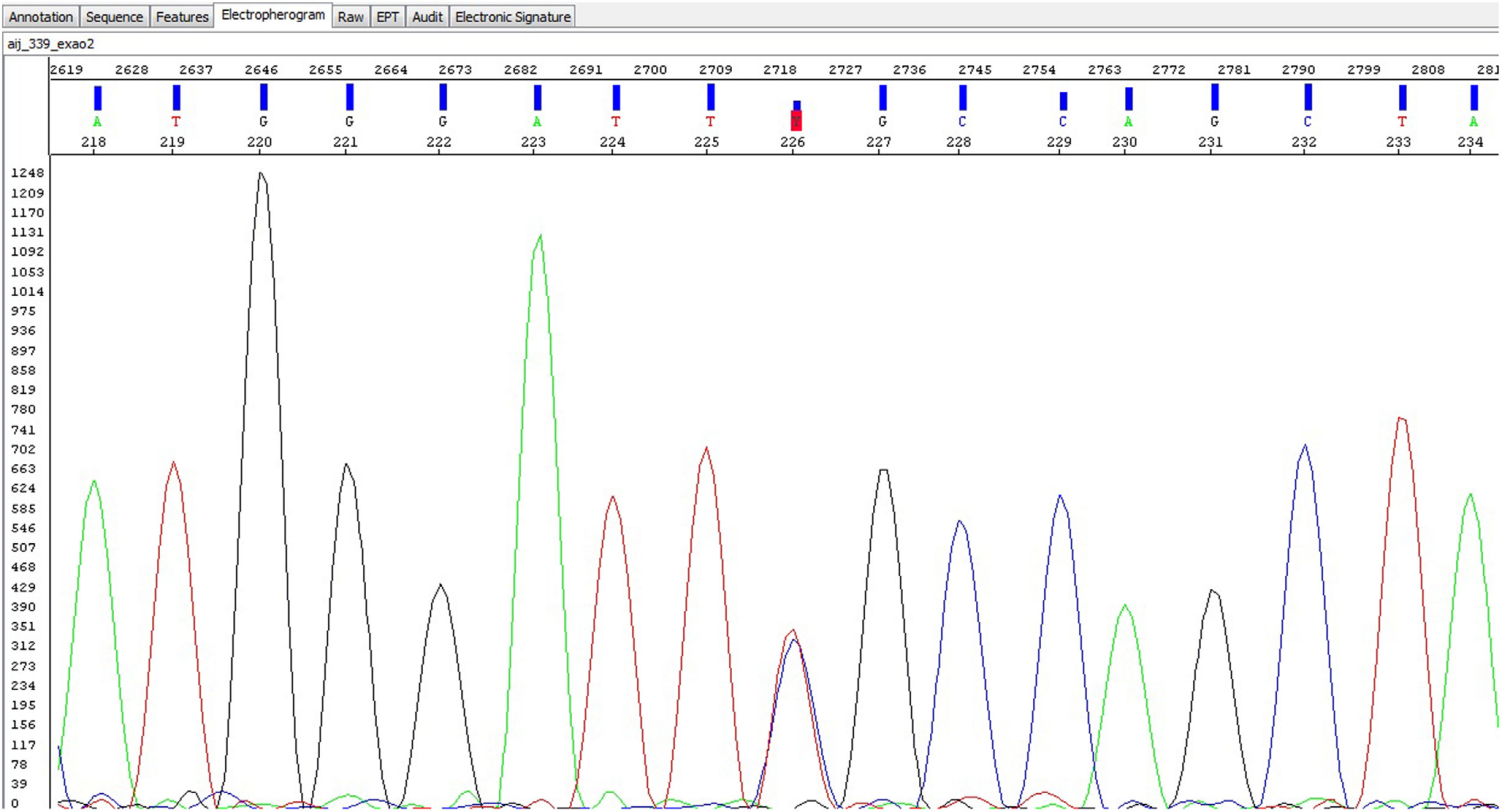
**Electropherogram of the R118C mutation**.

Mutations were identified in five unrelated patients (all females)—Table 2. Four patients had the alpha-Gal A gene mutation D313Y (GAT to TAT at cDNA nucleotide 937), and one patient presented the mutation R118C (a missense mutation)—electropherogram (Figure 1). The mutations were found in patients from five centers in four different cities (Oporto, Coimbra, Lisbon, and Faro), showing a non-clustered distribution of the alterations D313Y and R118C.

**Table 2:** **Clinical and demographic features of the patients with mutations**.

Patient	Mutation	Form of juvenile idiopathic arthritis	Associated conditions	Current age (years)	Age at disease onset (years)	Treatment
1	R118C	Persistent oligoarticular	Uveitis	6	3	MTX, NSAID
2	D313Y	Polyarticular RF−	–	21	5	MTX, NSAID
3	D313Y	Polyarticular RF−	Cervical Spine Involvement	19	4	ETA, MTX, NSAID
4	D313Y	Persistent oligoarticular	–	25	9	MTX, NSAID
5	D313Y	Persistent oligoarticular	Uveitis	9	8	MTX, NSAID

*ETA, etanercept; MTX, methotrexate; NSAID, non-steroid anti-inflammatory drugs; RF, rheumatoid factor*.

The patient with the R118C mutation is a 9-year-old girl who was diagnosed with oligoarticular JIA and uveitis when she was 3 years old. Joint involvement was restricted to knee episodes of arthritis, managed initially with intra-articular steroid injections and since 2010 with subcutaneous methotrexate. She never had skin lesions and GI symptoms, and her electrocardiogram was normal. Her labs were unremarkable, except for positive antinuclear antibodies at a titer of 1/160 and increased inflammatory markers. She had normal levels of plasma globotriaosylsphingosine (lyso-Gb3) (1.2 ng/mL, *N* < 1.8 ng/mL). Her family history is unremarkable for stroke at young age (<55 years), end-stage renal disease or premature death (before the fifth decade of life). Her mother suffers from Wolff–Parkinson–White (WPW) syndrome, and her father is type 2 diabetic non-insulin treated. One of her two sisters was born with hip dysplasia but with that exception her three siblings (aged 15, 16, and 18) are healthy.

The clinical history from the four patients with D313Y mutation was also revised. Patients were unremarkable for pain with neuropathic features, skin lesions, and GI symptoms. None of the JIA patients suffered from stroke, myocardiopathy, or chronic kidney disease. Their family members did not show any relevant finding, including chronic kidney disease, cardiac anomalies, and cerebrovascular events at young age.

## Discussion

To our knowledge, this was the first study in which FD was screened in a large cohort of JIA patients. No “classic” pathogenic FD mutations were found. The late-onset FD-associated mutation (R118C) was reported in a frequency of 0.34% (1 in 292 individuals) within this nationwide JIA cohort.

Several screenings of FD have been pursued in young adults with stroke, including one in a Portuguese population (19), in hemodialyzed patients (20) and in patients with hypertrophic cardiomyopathy (21). Our cohort is the youngest to be screened until the present moment, as far as we know. Screening of young populations for FD may become particularly important as some advocate initiation of ERT in children (22), although the international position paper (2015) adopt a more cautious view (23).

The mutational analysis through direct sequencing performed to screen for FD assured the same sensitivity for both genders, overcoming the limitations of alpha-Gal activity assays to diagnose FD in females.

The most commonly mutation found in our population was D313Y, which pathological significance for FD has been doubted, because in general D313Y patients do not present clinical manifestations typical of FD (24). Also, it was also proven that Gb3 does not accumulate in the lysosomes of D313Y patients (24).

R118C mutation was reported for the first time in a screening of 37,104 consecutive newborn males in Italy (25). R118C mutation was found in a neonate deficient in alpha-Gal A activity (double assay). This missense mutation had structural characteristics and *in vitro* overexpression levels similar to those of known later-onset missense mutations (25).

As our patient is a female and R188C is a mutation associated to late-onset FD, we cannot ascertain a final diagnosis of FD.

The patient and her family are under further clinical investigation. According to diagnostic criteria (15), there is no need to access enzymatic activity (as it can be normal in a female patient). It should be verified if classical symptoms develop or if her parents have definite FD diagnosis carrying the same *GLA* mutation.

The patient’s mother suffers from WPW syndrome, and an association of WPW with autosomal dominant familial hypertrophic cardiomyopathy has been described (26). However, there are no reports of a direct association of FD and WPW.

A low threshold for FD in JIA patients should be warranted, as pain maybe an initial manifestation during the first decades of life (7, 8). Also, a family history of strokes at young age, early onset chronic kidney disease, or myocardiopathy should further elevate the clinical suspicion.

Late manifestations of FD, namely, poststroke status, end-stage renal disease, and hypertrophic myocardiopathy, are associated with high morbidity and mortality. On the contrary, early diagnosis of FD can lead to implementation of a specific therapy and allow for the study of their families and respective early treatment if justified. Therefore, finding strategies to screen younger populations, in whom such events could be prevented, seems crucial.

Cost-effectiveness of population screening, even in high-risk groups is difficult to estimate, considering the high direct and indirect costs of this disease in late stages, but also the high cost of enzyme replacement therapies.

Further studies should assess optimal ways of defining high-risk young populations, maybe by combining family history with the presence of symptoms, such as those occurring in JIA.

## Author Contributions

MG and AFM wrote the main manuscript text; AM and OS did the laboratory work; MG, AFM, JM-G, MS, PE, CR, and IB collected clinical data; JF and HC designed the research project. All authors reviewed the manuscript and approved the final version.

## Conflict of Interest Statement

The authors declare that the research was conducted in the absence of any commercial or financial relationships that could be construed as a potential conflict of interest.
