# Supplementary material for: Genetic Screening of Mutations Associated with Fabry Disease in a Nationwide Cohort of Juvenile Idiopathic Arthritis Patients
Source: Front Med (Lausanne). 2017 Mar 1;4:12. doi: 10.3389/fmed.2017.00012 (PMC5331034; doi:10.3389/fmed.2017.00012)
Supplement: Supplementary file 1 [file Table_1.docx]

**Table 1** – Diagnostic Criteria for Fabry disease, adapted from Smid *et al*.

| Males | Females |
| --- | --- |
| GLA mutation + AGAL_A deficiency of <5% of mean reference value in leukocytes | GLA mutation + normal or deficient AGAL-A in leukocytes |
| **+** A or B or C | |
| **A** ≥1 characteristic FD s/s (neuropathic pain, cornea verticillata or clustered angiokeratoma) | |
| **B** an increase of plasma (lyso)Gb3 (within range of males with definite FD diagnosis) | |
| **C** a family member with a definite FD diagnosis carrying the same GLA mutation | |
